# Supplementary material for: The Challenge of Diffusion Magnetic Resonance Imaging in Cerebral Palsy: A Proposed Method to Identify White Matter Pathways
Source: Brain Sci. 2023 Sep 29;13(10):1386. doi: 10.3390/brainsci13101386 (PMC10605121; doi:10.3390/brainsci13101386)
Supplement: Supplementary file 1 [file brainsci-13-01386-s001.zip › brainsci-2592747-supplementary.pdf]

Table S1. Presence (✓) or absence (✗) of the tracts for each participant. L and R respectively stands for left and right.

		S1	S2	S3	S4	S5	S6	S7	S8
Anterior commissure		✓	✓	✓	✓	✓	✓	✓	✓
Arcuate fasciculus	L	✓	✓	✓	✓	✗	✓	✓	✓
	R	✓	✓	✗	✓	✓	✗	✓	✓
Corpus callosum, anterior part of frontal lobe		✓	✓	✓	✓	✓	✗	✓	✓
Corpus callosum, posterior part of frontal lobe		✓	✓	✓	✓	✓	✗	✓	✓
Corpus callosum, occipital lobe		✓	✓	✓	✓	✓	✗	✓	✓
Corpus callosum, parietal lobe		✓	✓	✓	✓	✓	✗	✓	✓
Corpus callosum, pre-post central gyri		✓	✓	✓	✓	✓	✗	✓	✓
Corpus callosum, temporal lobe		✓	✗	✓	✓	✓	✗	✗	✗
Cingulum	L	✓	✓	✗	✓	✓	✓	✓	✓
	R	✓	✓	✓	✓	✓	✗	✓	✓
Frontal aslant tract	L	✓	✗	✓	✓	✗	✓	✓	✓
	R	✓	✓	✗	✓	✓	✗	✓	✓
Fronto pontine tract	L	✓	✓	✓	✓	✓	✓	✓	✓
	R	✓	✓	✓	✓	✓	✗	✓	✓
Fornix	L	✓	✓	✓	✓	✓	✓	✓	✓
	R	✓	✓	✓	✓	✓	✗	✓	✓
Inferior fronto-occipital fasciculus	L	✓	✓	✓	✓	✓	✓	✓	✓
	R	✓	✓	✓	✓	✓	✗	✓	✓
Inferior longitudinal fasciculus	L	✓	✓	✓	✓	✓	✓	✓	✓
	R	✓	✓	✓	✓	✓	✗	✓	✓
Middle cerebellar peduncle		✓	✓	✓	✓	✓	✓	✓	✓
Middle longitudinal fascicle	L	✓	✓	✓	✓	✓	✓	✓	✓
	R	✓	✓	✗	✓	✓	✓	✓	✓
Optic radiation and Meyer’s loop	L	✓	✓	✓	✓	✓	✓	✓	✓
	R	✓	✓	✓	✓	✓	✗	✓	✓
Posterior comissure		✓	✓	✓	✓	✗	✓	✓	✓
Parieto-occipito pontine tract	L	✓	✓	✓	✓	✓	✓	✓	✓
	R	✓	✓	✗	✓	✓	✗	✓	✓
Pyramidal tract	L	✓	✓	✓	✓	✓	✓	✓	✓
	R	✓	✓	✓	✓	✓	✗	✓	✓
Superior cerebellar peduncle	L	✓	✓	✓	✓	✓	✓	✓	✓
	R	✓	✓	✓	✓	✓	✓	✓	✓
Superior longitudinal fasciculus	L	✓	✓	✓	✓	✗	✓	✓	✓
	R	✓	✓	✗	✓	✓	✓	✓	✓
Uncinate fasciculus	L	✓	✓	✓	✓	✓	✓	✓	✓
	R	✓	✓	✓	✓	✓	✗	✓	✓

Table S2. Overall results for left and right CST tracts (lCST, rCST) for the participants. The mean value (and the standard deviation) is presented for A) the volume and DTI metrics (i.e., FA, MD, RD, AD), B) the fODF metrics (i.e., AFD total and NuFO) and C) the NODDI metrics (i.e., OD, ICVF, ISOVF). The AI is computed between the controlesional and the ipsilesional side for each metric.

A) Volume and DTI metrics

	**Volume (in mm³)**			**FA**			**MD**			**RD**			**AD**		
	lCST	rCST	AI	lCST	rCST	AI	lCST	rCST	AI	lCST	rCST	AI	lCST	rCST	AI
**S1**	59061	42190	**0.17**	0.58<br>(0.17)	0.54<br>(0.17)	**0.04**	0.0007<br>(0.0002)	0.0008<br>(0.0003)	**-0.06**	0.0005<br>(0.0002)	0.0005<br>(0.0003)	**-0.09**	0.0013<br>(0.0003)	0.0014<br>(0.0004)	**-0.04**
**S2**	32783	73372	**-0.38**	0.46<br>(0.17)	0.53<br>(0.17)	**-0.07**	0.0009<br>(0.0003)	0.0007<br>(0.0002)	**0.12**	0.0007<br>(0.0003)	0.0005<br>(0.0002)	**0.16**	0.0014<br>(0.0004)	0.0012<br>(0.0003)	**0.09**
**S3**	69756	28592	**0.42**	0.48<br>(0.18)	0.52<br>(0.19)	**-0.03**	0.0008<br>(0.0004)	0.0009<br>(0.0003)	**-0.02**	0.0006<br>(0.0004)	0.0006<br>(0.0003)	**0.02**	0.0013<br>(0.0005)	0.0014<br>(0.0004)	**-0.05**
**S4**	72236	70685	**0.01**	0.51<br>(0.17)	0.52<br>(0.17)	**-0.01**	0.0008<br>(0.0004)	0.0008<br>(0.0003)	**0.01**	0.0005<br>(0.0004)	0.0005<br>(0.0003)	**0.02**	0.0013<br>(0.0005)	0.0013<br>(0.0004)	**0.01**
**S5**	50380	70970	**-0.17**	0.49<br>(0.16)	0.53<br>(0.18)	**-0.04**	0.0009<br>(0.0003)	0.0008<br>(0.0002)	**0.06**	0.0006<br>(0.0003)	0.0005<br>(0.0002)	**0.09**	0.0014<br>(0.0004)	0.0013<br>(0.0003)	**0.03**
**S6**	70482	No data		0.48<br>(0.17)	No data		0.0008<br>(0.0005)	No data		0.0006<br>(0.0004)	No data		0.0013<br>(0.0006)	No data	
**S7**	72841	65878	**0.05**	0.54<br>(0.17)	0.51<br>(0.17)	**0.02**	0.0007<br>(0.0002)	0.0008<br>(0.0002)	**-0.03**	0.0005<br>(0.0002)	0.0005<br>(0.0002)	**0.00**	0.0012<br>(0.0003)	0.0013<br>(0.0004)	**-0.04**
**S8**	57038	32673	**0.27**	0.48<br>(0.16)	0.43<br>(0.15)	**0.06**	0.0008<br>(0.0003)	0.0010<br>(0.0004)	**-0.09**	0.0006<br>(0.0003)	0.0007<br>(0.0004)	**0.07**	0.0012<br>(0.0004)	0.0014<br>(0.0005)	**-0.08**

B) fODF metrics

	AFDtotal			NuFO		
	lCST	rCST	AI	lCST	rCST	AI
**S1**	0.36 (0.058)	0.34 (0.52)	**0.03**	1.29 (0.53)	1.27 (0.51)	**0.01**
**S2**	0.34 (0.049)	0.40 (0.062)	**-0.07**	1.42 (0.66)	1.57 (0.67)	**-0.05**
**S3**	0.36 (0.067)	0.34 (0.051)	**0.03**	1.54 (0.70)	1.26 (0.53)	**0.10**
**S4**	0.35 (0.051)	0.36 (0.055)	**-0.01**	1.50 (0.70)	1.51 (0.68)	**0.00**
**S5**	0.31 (0.04)	0.34 (0.06)	**-0.04**	1.39 (0.60)	1.38 (0.61)	**0.00**
**S6**	0.34 (0.06)	No data		1.52 (0.66)	No data	
**S7**	0.91 (0.40)	0.87 (0.38)	**-0.02**	1.30 (0.52)	1.25 (0.50)	**-0.02**
**S8**	0.75 (0.32)	0.70 (0.35)	**0.03**	1.37 (0.60)	1.22 (0.54)	**0.06**

C) NODDI metrics

	OD			ICVF			ISOVF		
	lCST	rCST	AI	lCST	rCST	AI	lCST	rCST	AI
**S1**	0.15<br>(0.12)	0.15<br>(0.12)	**0.00**	0.65<br>(0.11)	0.60<br>(0.12)	**0.04**	0.10<br>(0.12)	0.14<br>(0.16)	**-0.09**
**S2**	0.17<br>(0.14)	0.19<br>(0.12)	**0.07**	0.52<br>(0.15)	0.67<br>(0.12)	**0.13**	0.14<br>(0.18)	0.10<br>(0.10)	**0.16**
**S3**	0.20<br>(0.15)	0.14<br>(0.13)	**0.16**	0.63<br>(0.16)	0.53<br>(0.14)	**0.08**	0.15<br>(0.19)	0.11<br>(0.17)	**0.02**
**S4**	0.19<br>(0.14)	0.18<br>(0.13)	**0.03**	0.63<br>(0.13)	0.62<br>(0.12)	**0.00**	0.13<br>(0.15)	0.12<br>(0.14)	**0.02**
**S5**	0.16<br>(0.13)	0.16<br>(0.12)	**0.01**	0.55<br>(0.15)	0.61<br>(0.12)	**0.06**	0.14<br>(0.16)	0.10<br>(0.14)	**0.09**
**S6**	0.20<br>(0.15)	No<br>data		0.63<br>(0.16)	No data		0.15<br>(0.18)	No data	
**S7**	0.17<br>(0.12)	0.17<br>(0.13)	**-0.01**	0.64<br>(0.11)	0.58<br>(0.12)	**0.05**	0.10<br>(0.11)	0.09<br>(0.14)	**0.05**
**S8**	0.20<br>(0.13)	0.18<br>(0.14)	**-0.04**	0.59<br>(0.13)	0.50<br>(0.18)	**-0.08**	0.11<br>(0.16)	0.16<br>(0.21)	**0.19**

Table S3. Overall results for the six sections of the corpus callosum (CC_Fr1 and CC_Fr2: Frontal projections, CC_Pr_Po: Pre- and post-central gyri, CC_Pa: parietal, CC_Te: Temporal, CC_Oc: occipital) for the participants. The mean value (and the standard deviation) is presented for the FA, MD, RD, AD, AFD totale and NuFO metrics. The total volume is indicated for each tract in mm³.

A) Volume and DTI metrics

	**Volume (in mm³)**						**FA**					
	CC_Fr1	CC_Fr2	CC_Pr_Po	CC_Pa	CC_Te	CC_Oc	CC_Fr1	CC_Fr2	CC_Pr_Po	CC_Pa	CC_Te	CC_Oc
**S1**	58388	51361	45769	53679	5167	51133	0.49 (0.17)	0.44 (0.15)	0.51 (0.17)	0.53 (0.19)	0.53 (0.18)	0.56 (0.19)
**S2**	37228	51636	47566	31578	1761	42337	0.51 (0.19)	0.48 (0.18)	0.51 (0.18)	0.57 (0.21)	0.52 (0.18)	0.64 (0.21)
**S3**	41769	3808	24103	11242	11151	15582	0.44 (0.17)	0.43 (0.15)	0.43 (0.14)	0.47 (0.19)	0.51 (0.14)	0.59 (0.20)
**S4**	69411	81435	69396	56006	7627	35025	0.46 (0.17)	0.49 (0.18)	0.50 (0.17)	0.52 (0.19)	0.57 (0.17)	0.61 (0.21)
**S5**	37610	39391	60558	62447	1607	54212	0.45 (0.17)	0.39 (0.14)	0.49 (0.17)	0.52 (0.18)	0.49 (0.18)	0.58 (0.19)
**S6**	No data						No data					
**S7**	85177	120929	81646	72994	No data	53327	0.47 (0.18)	0.49 (0.18)	0.52 (0.18)	0.53 (0.20)	No data	0.55 (0.21)
**S8**	54560	14458	27568	48199	223	30579	0.42 (0.17)	0.31 (0.13)	0.42 (0.18)	0.45 (0.19)	0.39 (0.17)	0.44 (0.17)

	MD						RD						AD					
	CC_Fr1	CC_Fr2	CC_Pr_Po	CC_Pa	CC_Te	CC_Oc	CC_Fr1	CC_Fr2	CC_Pr_Po	CC_Pa	CC_Te	CC_Oc	CC_Fr1	CC_Fr2	CC_Pr_Po	CC_Pa	CC_Te	CC_Oc
**S1**	0.0008 (0.0002)	0.0009 (0.0003)	0.0009 (0.0004)	0.0008 (0.0002)	0.0008 (0.0002)	0.0008 (0.0003)	0.0006 (0.0002)	0.0007 (0.0003)	0.0006 (0.0003)	0.0005 (0.0002)	0.0005 (0.0002)	0.0005 (0.0003)	0.0013 (0.0003)	0.0014 (0.0004)	0.0014 (0.0005)	0.0014 (0.0004)	0.0014 (0.0004)	0.0015 (0.0004)
**S2**	0.0008 (0.0002)	0.0008 (0.0003)	0.0009 (0.0004)	0.0008 (0.0001)	0.0009 (0.0003)	0.0008 (0.0002)	0.0005 (0.0002)	0.0006 (0.0002)	0.0006 (0.0003)	0.0005 (0.0002)	0.0006 (0.0003)	0.0004 (0.0002)	0.0013 (0.0004)	0.0013 (0.0004)	0.0014 (0.0006)	0.0014 (0.0003)	0.0014 (0.0005)	0.0015 (0.0004)
**S3**	0.0009 (0.0002)	0.0011 (0.0005)	0.0011 (0.0005)	0.0009 (0.0002)	0.0009 (0.0003)	0.0008 (0.0002)	0.0007 (0.0002)	0.0008 (0.0004)	0.0008 (0.0005)	0.0006 (0.0002)	0.0006 (0.0003)	0.0005 (0.0002)	0.0014 (0.0003)	0.0016 (0.0006)	0.0016 (0.0007)	0.0014 (0.0004)	0.0015 (0.0004)	0.0015 (0.0004)
**S4**	0.0009 (0.0002)	0.0008 (0.0003)	0.0009 (0.0003)	0.0008 (0.0002)	0.0008 (0.0002)	0.0008 (0.0001)	0.0006 (0.0002)	0.0006 (0.0003)	0.0006 (0.0003)	0.0005 (0.0002)	0.0005 (0.0003)	0.0005 (0.0002)	0.0014 (0.0004)	0.0013 (0.0004)	0.0014 (0.0005)	0.0014 (0.0003)	0.0014 (0.0004)	0.0015 (0.0003)
**S5**	0.0009 (0.0002)	0.0010 (0.0003)	0.0009 (0.0003)	0.0008 (0.0002)	0.0009 (0.0002)	0.0008 (0.0002)	0.0006 (0.0002)	0.0007 (0.0003)	0.0006 (0.0003)	0.0006 (0.0002)	0.0006 (0.0003)	0.0005 (0.0002)	0.0014 (0.0004)	0.0014 (0.0005)	0.0014 (0.0005)	0.0014 (0.0004)	0.0014 (0.0004)	0.0015 (0.0004)
**S6**	No data						No data						No data					
**S7**	0.0008 (0.0002)	0.0008 (0.0002)	0.0008 (0.0003)	0.0008 (0.0002)	No data	0.0009 (0.0004)	0.0006 (0.0002)	0.0006 (0.0002)	0.0006 (0.0003)	0.0005 (0.0002)	No data	0.0006 (0.0004)	0.0013 (0.0003)	0.0013 (0.0004)	0.0013 (0.0005)	0.0013 (0.0003)	No data	0.0016 (0.0006)
**S8**	0.0009 (0.0002)	0.0012 (0.0005)	0.0010 (0.0003)	0.0009 (0.0002)	0.0012 (0.0006)	0.0010 (0.0004)	0.0007 (0.0002)	0.0010 (0.0005)	0.0008 (0.0003)	0.0006 (0.0002)	0.0009 (0.0006)	0.0008 (0.0004)	0.0014 (0.0004)	0.0015 (0.0006)	0.0015 (0.0005)	0.0014 (0.0004)	0.0017 (0.0006)	0.0016 (0.0005)

B) fODF metrics

	AFDtotal						NuFO					
	CC_Fr1	CC_Fr2	CC_Pr_Po	CC_Pa	CC_Te	CC_Oc	CC_Fr1	CC_Fr2	CC_Pr_Po	CC_Pa	CC_Te	CC_Oc
**S1**	0.25	0.28	0.32	0.32	0.32	0.33	1.11	1.14	1.27	1.22	1.38	1.19
	(0.02)	(0.03)	(0.04)	(0.04)	(0.03)	(0.05)	(0.46)	(0.47)	(0.55)	(0.52)	(0.58)	(0.48)
**S2**	0.26	0.30	0.32	0.32	0.32	0.36	1.19	1.30	1.39	1.29	1.43	1.24
	(0.03)	(0.03)	(0.04)	(0.03)	(0.04)	(0.03)	(0.53)	(0.69)	(0.61)	(0.57)	(0.75)	(0.55)
**S3**	0.21	0.28	0.30	0.30	0.31	0.31	0.81	1.23	1.32	1.32	1.37	1.19
	(0.06)	(0.04)	(0.05)	(0.03)	(0.04)	(0.03)	(0.56)	(0.51)	(0.57)	(0.59)	(0.58)	(0.45)
**S4**	0.26	0.33	0.35	0.33	0.34	0.34	1.11	1.39	1.48	1.37	1.53	1.25
	(0.05)	(0.04)	(0.04)	(0.03)	(0.03)	(0.04)	(0.62)	(0.63)	(0.70)	(0.64)	(0.72)	(0.55)
**S5**	0.23	0.25	0.29	0.29	0.30	0.32	1.16	1.30	1.35	1.31	1.51	1.31
	(0.04)	(0.03)	(0.03)	(0.02)	(0.03)	(0.04)	(0.55)	(0.58)	(0.59)	(0.56)	(0.66)	(0.57)
**S6**	No data						No data					
**S7**	0.63	0.70	0.87	0.80	No data	0.95	1.02	1.15	1.22	1.20	No data	0.95
	(0.32)	(0.33)	(0.38)	(0.40)		(0.45)	(0.45)	(0.45)	(0.48)	(0.47)		(0.45)
**S8**	0.63	0.53	0.72	0.68	0.63	0.72	1.17	1.16	1.27	1.35	1.26	1.24
	(0.31)	(0.25)	(0.32)	(0.30)	(0.40)	(0.32)	(0.59)	(0.58)	(0.56)	(0.61)	(0.65)	(0.54)

C) NODDI metrics

	OD						ICVF						ISOVF					
	CC_Fr1	CC_Fr2	CC_Pr_Po	CC_Pa	CC_Te	CC_Oc	CC_Fr1	CC_Fr2	CC_Pr_Po	CC_Pa	CC_Te	CC_Oc	CC_Fr1	CC_Fr2	CC_Pr_Po	CC_Pa	CC_Te	CC_Oc
**S1**	0.17 (0.11)	0.17 (0.13)	0.14 (0.14)	0.15 (0.13)	0.16 (0.13)	0.16 (0.13)	0.55 (0.13)	0.52 (0.12)	0.59 (0.14)	0.59 (0.12)	0.60 (0.13)	0.60 (0.14)	0.12 (0.14)	0.16 (0.20)	0.16 (0.21)	0.13 (0.15)	0.14 (0.16)	0.15 (0.18)
**S2**	0.18 (0.13)	0.18 (0.14)	0.11 (0.15)	0.15 (0.13)	0.17 (0.15)	0.17 (0.11)	0.61 (0.15)	0.58 (0.12)	0.60 (0.13)	0.61 (0.11)	0.63 (0.15)	0.64 (0.13)	0.13 (0.15)	0.15 (0.17)	0.17 (0.21)	0.10 (0.11)	0.18 (0.20)	0.11 (0.12)
**S3**	0.19 (0.15)	0.16 (0.16)	0.12 (0.18)	0.16 (0.14)	0.17 (0.14)	0.15 (0.12)	0.54 (0.14)	0.54 (0.20)	0.55 (0.19)	0.50 (0.12)	0.54 (0.17)	0.59 (0.15)	0.18 (0.16)	0.24 (0.29)	0.26 (0.29)	0.13 (0.17)	0.16 (0.18)	0.14 (0.14)
**S4**	0.18 (0.14)	0.17 (0.13)	0.12 (0.15)	0.15 (0.12)	0.16 (0.12)	0.14 (0.11)	0.54 (0.13)	0.57 (0.13)	0.59 (0.13)	0.56 (0.14)	0.60 (0.15)	0.61 (0.15)	0.16 (0.16)	0.13 (0.17)	0.15 (0.20)	0.12 (0.13)	0.13 (0.14)	0.12 (0.10)
**S5**	0.16 (0.14)	0.18 (0.14)	0.13 (0.14)	0.14 (0.12)	0.15 (0.13)	0.17 (0.12)	0.45 (0.12)	0.45 (0.13)	0.54 (0.14)	0.53 (0.12)	0.54 (0.14)	0.59 (0.15)	0.10 (0.14)	0.15 (0.20)	0.14 (0.19)	0.11 (0.13)	0.13 (0.17)	0.13 (0.15)
**S6**	No data						No data						No data					
**S7**	0.18 (0.13)	0.18 (0.13)	0.16 (0.14)	0.16 (0.13)	No data	0.14 (0.16)	0.50 (0.12)	0.53 (0.12)	0.56 (0.12)	0.55 (0.14)	No data	0.55 (0.19)	0.10 (0.13)	0.09 (0.15)	0.11 (0.18)	0.08 (0.11)	No data	0.16 (0.23)
**S8**	0.19 (0.14)	0.26 (0.17)	0.19 (0.15)	0.19 (0.14)	0.22 (0.23)	0.17 (0.15)	0.50 (0.14)	0.52 (0.23)	0.56 (0.22)	0.53 (0.16)	0.49 (0.19)	0.53 (0.19)	0.18 (0.17)	0.30 (0.32)	0.26 (0.26)	0.16 (0.16)	0.32 (0.32)	0.22 (0.25)

Table S4. Overall results for left and right ML tracts (lML, rML) for the participants. The mean value (and the standard deviation) is presented for the FA, MD, RD, AD, AFD totale and NuFO metrics. The total volume is indicated for each tract in mm^3. The AI is computed between the controlesional and the ipsilesional side for each metric.

A) Volume and DTI metrics

	Volume (in mm^3)			FA			MD			RD			AD		
	lML	rML	AI	lML	rML	AI	lML	rML	AI	lML	rML	AI	lML	rML	AI
**S1**	9408	6359	**0.19**	0.54	0.53	**0.00**	0.0007	0.0008	**-0.04**	0.0005	0.0005	**-0.04**	0.0012	0.0013	**-0.04**
				(0.15)	(0.15)		(0.0002)	(0.0003)		(0.0002)	(0.0003)		(0.0003)	(0.0004)	
**S2**	7087	13907	**0.32**	0.46	0.51	**0.06**	0.0009	0.0007	**-0.11**	0.0006	0.0005	**-0.14**	0.0014	0.0012	**-0.09**
				(0.15)	(0.14)		(0.0003)	(0.0001)		(0.0003)	(0.0001)		(0.0004)	(0.0002)	
**S3**	13129	7399	**0.28**	0.46	0.49	**-0.03**	0.0008	0.0010	**-0.13**	0.0006	0.0007	**-0.11**	0.0012	0.0014	**-0.14**
				(0.15)	(0.14)		(0.0002)	(0.0004)		(0.0004)	(0.0003)		(0.0003)	(0.0005)	
**S4**	9939	11697	**-0.08**	0.51	0.53	**-0.02**	0.0008	0.0007	**0.01**	0.0005	0.0005	**0.02**	0.0012	0.0012	**0.00**
				(0.14)	(0.14)		(0.0002)	(0.0002)		(0.0002)	(0.0002)		(0.0003)	(0.0002)	
**S5**	9526	9162	**-0.0.2**	0.50	0.51	**0.01**	0.0008	0.0007	**-0.05**	0.0006	0.0005	**-0.06**	0.0013	0.0012	**-0.04**
				(0.15)	(0.14)		(0.0002)	(0.0002)		(0.0002)	(0.0002)		(0.0003)	(0.0003)	
**S6**		No data			No data			No data			No data			No data	
**S7**	15187	9546	**0.23**	0.54	0.56	**-0.02**	0.0007	0.0007	**0.00**	0.0005	0.0005	**0.00**	0.0012	0.0013	**-0.04**
				(0.15)	(0.14)		(0.0001)	(0.0001)		(0.0001)	(0.0002)		(0.0002)	(0.0002)	
**S8**	7189	4296	**0.25**	0.47	0.40	**0.08**	0.0008	0.0010	**-0.11**	0.0006	0.0008	**-0.14**	0.0013	0.0014	**-0.04**
				(0.15)	(0.16)		(0.0002)	(0.0004)		(0.0003)	(0.0004)		(0.0003)	(0.0005)	

B) fODF metrics

	AFDtotal			NuFO		
	lML	rML	AI	lML	rML	AI
**S1**	0.80 (0.31)	0.86 (0.39)	**-0.03**	1.28 (0.51)	1.25 (0.50)	**0.19**
**S2**	0.74 (0.33)	0.76 (0.30)	**0.01**	1.35 (0.59)	1.61 (0.68)	**0.09**
**S3**	0.66 (0.27)	0.84 (0.29)	**-0.12**	1.57 (0.67)	1.24 (0.48)	**0.12**
**S4**	0.73 (0.28)	0.75 (0.31)	**-0.01**	1.35 (0.57)	1.42 (0.62)	**-0.02**
**S5**	0.69 (0.31)	0.67 (0.27)	**-0.04**	1.33 (0.57)	1.41 (0.60)	**0.03**
**S6**	No data			No data		
**S7**	0.87 (0.33)	0.93 (0.33)	**-0.03**	1.23 (0.46)	1.17 (0.41)	**0.03**
**S8**	0.75 (0.28)	0.66 (0.36)	**0.06**	1.39 (0.56)	1.24 (0.51)	**0.06**

C) NODDI metrics

	OD			ICVF			ISOVF		
	lML	rML	AI	lML	rML	AI	lML	rML	AI
**S1**	0.16 (0.10)	0.15 (0.11)	**0.03**	0.62 (0.10)	0.60 (0.11)	**0.01**	0.10 (0.13)	0.13 (0.16)	**-0.14**
**S2**	0.16 (0.11)	0.19 (0.10)	**0.09**	0.51 (0.14)	0.64 (0.11)	**0.12**	0.13 (0.16)	0.09 (0.10)	**-0.17**
**S3**	0.20 (0.11)	0.11 (0.10)	**0.27**	0.60 (0.12)	0.52 (0.16)	**0.08**	0.11 (0.13)	0.19 (0.23)	**-0.26**
**S4**	0.17 (0.10)	0.16 (0.09)	**0.02**	0.61 (0.11)	0.61 (0.10)	**0.00**	0.12 (0.13)	0.10 (0.11)	**0.05**
**S5**	0.15 (0.10)	0.17 (0.10)	**0.06**	0.54 (0.12)	0.58 (0.10)	**0.03**	0.13 (0.13)	0.08 (0.11)	**-0.21**
**S6**	No data			No data			No data		
**S7**	0.16 (0.10)	0.14 (0.10)	**0.07**	0.62 (0.10)	0.57 (0.10)	**0.04**	0.09 (0.10)	0.07 (0.10)	**0.13**
**S8**	0.19 (0.10)	0.19 (0.14)	**0.00**	0.56 (0.12)	0.49 (0.19)	**0.07**	0.12 (0.17)	0.18 (0.24)	**-0.20**

Table S5. Overall results for left and right FPC tracts (lFPC, rFPC) for the participants. The mean value (and the standard deviation) is presented for the FA, MD, RD, AD, AFD totale and NuFO metrics. The total volume is indicated for each tract in mm³. The AI is computed between the controlesional and the ipsilesional side for each metric.

A) Volume and DTI metrics

	**Volume (in mm³)**			**FA**			**MD**			**RD**			**AD**		
	lFPC	rFPC	AI	lFPC	rFPC	AI	lFPC	rFPC	AI	lFPC	rFPC	AI	lFPC	rFPC	AI
**S1**	10974	2620	**0.61**	0.53 (0.17)	0.50 (0.17)	**0.03**	0.0007 (0.0001)	0.0008 (0.0002)	**-0.03**	0.0005 (0.0001)	0.0005 (0.0002)	**-0.04**	0.0012 (0.0003)	0.0012 (0.0003)	**-0.01**
**S2**	185	1542	**0.79**	0.46 (0.14)	0.52 (0.18)	**0.07**	0.0009 (0.0004)	0.0007 (8.3E-05)	**-0.13**	0.0007 (0.0004)	0.0005 (0.0001)	**-0.18**	0.0014 (0.0005)	0.0012 (0.0002)	**-0.09**
**S3**	7138	13616	**-0.31**	0.48 (0.17)	0.52 (0.17)	**-0.04**	0.0008 (0.0004)	0.0009 (0.0004)	**-0.03**	0.0006 (0.0004)	0.0006 (0.0004)	**0.00**	0.0013 (0.0005)	0.0014 (0.0005)	**-0.05**
**S4**	7463	15095	**-0.34**	0.49 (0.17)	0.47 (0.16)	**0.02**	0.0008 (0.0003)	0.0008 (0.0003)	**0.00**	0.0005 (0.0003)	0.0006 (0.0003)	**-0.01**	0.0012 (0.0004)	0.0012 (0.0004)	**0.01**
**S5**	159	4557	**0.93**	0.40 (0.17)	0.49 (0.18)	**0.09**	0.0001 (0.0006)	0.0008 (0.0002)	**-0.13**	0.0008 (0.0006)	0.0005 (0.0002)	**-0.20**	0.0014 (0.0007)	0.0012 (0.0003)	**-0.07**
**S6**		No data			No data			No data			No data			No data	
**S7**	1582	2183	**-0.16**	0.49 (0.15)	0.47 (0.16)	**0.02**	0.0008 (0.0002)	0.0008 (0.0004)	**0.0**	0.0005 (0.0002)	0.0006 (0.0004)	**-0.09**	0.0012 (0.0003)	0.0013 (0.0005)	**-0.04**
**S8**	1627	444	**0.57**	0.45 (0.17)	0.39 (0.18)	**0.07**	0.0008 (0.0001)	0.0015 (0.0014)	**-0.30**	0.0006 (0.0002)	0.0013 (0.0012)	**-0.37**	0.0012 (0.0003)	0.0021 (0.0017)	**-0.27**

B) fODF metrics

	AFDtotal			NuFO		
	lFPC	rFPC	AI	lFPC	rFPC	AI
**S1**	0.73	0.73	**0.01**	1.33	1.26	**0.03**
	(0.37)	(0.42)		(0.56)	(0.52)	
**S2**	0.65	0.73	**0.05**	1.56	1.57	**0.00**
	(0.37)	(0.44)		(0.76)	(0.74)	
**S3**	0.69	0.76	**-0.05**	1.56	1.30	**0.09**
	(0.37)	(0.37)		(0.72)	(0.57)	
**S4**	0.66	0.62	**0.03**	1.64	1.65	**0.00**
	(0.34)	(0.29)		(0.77)	(0.78)	
**S5**	0.55	0.65	**0.09**	1.55	1.59	**0.01**
	(0.31)	(0.35)		(0.86)	(0.71)	
**S6**	No data			No data		
**S7**	0.70	0.70	**0.0**	1.29	1.27	**0.01**
	(0.37)	(0.38)		(0.53)	(0.52)	
**S8**	0.62	0.57	**0.04**	1.34	1.01	**0.14**
	(0.37)	(0.40)		(0.65)	(0.63)	

C) NODDI metrics

	OD			ICVF			ISOVF		
	lFPC	rFPC	AI	lFPC	rFPC	AI	lFPC	rFPC	AI
**S1**	0.17 (0.12)	0.18 (0.12)	**-0.03**	0.65 (0.12)	0.61 (0.12)	**0.03**	0.11 (0.10)	0.13 (0.13)	**-0.08**
**S2**	0.17 (0.13)	0.20 (0.13)	**0.08**	0.55 (0.16)	0.69 (0.12)	**0.11**	0.17 (0.22)	0.12 (0.08)	**-0.17**
**S3**	0.20 (0.15)	0.15 (0.12)	**0.14**	0.62 (0.17)	0.58 (0.18)	**0.03**	0.15 (0.19)	0.16 (0.20)	**-0.03**
**S4**	0.20 (0.14)	0.20 (0.13)	**0.00**	0.62 (0.13)	0.61 (0.12)	**0.01**	0.12 (0.14)	0.11 (0.15)	**0.04**
**S5**	0.27 (0.24)	0.19 (0.12)	**-0.17**	0.49 (0.23)	0.60 (0.13)	**0.10**	0.18 (0.28)	0.11 (0.13)	**-0.24**
**S6**		No data			No data			No data	
**S7**	0.20 (0.12)	0.20 (0.13)	**0.0**	0.63 (0.13)	0.60 (0.15)	**0.02**	0.11 (0.14)	0.12 (0.20)	**-0.04**
**S8**	0.22 (0.13)	0.29 (0.30)	**-0.14**	0.57 (0.12)	0.48 (0.29)	**0.09**	0.10 (0.12)	0.32 (0.35)	**-0.52**

Table S6. Overall results for left and right CTF tracts (lCTF, rCTF) for the participants. The mean value (and the standard deviation) is presented for the FA, MD, RD, AD, AFD totale and NuFO metrics. The total volume is indicated for each tract in mm³. The AI is computed between the controlesional and the ipsilesional side for each metric.

A) Volume and DTI metrics

	**Volume (in mm³)**			**FA**			**MD**			**RD**			**AD**		
	lCTF	rCTF	AI	lCTF	rCTF	AI	lCTF	rCTF	AI	lCTF	rCTF	AI	lCTF	rCTF	AI
**S1**	11537	1244	**0.81**	0.45	0.41	**0.04**	0.0008	0.0011	**-0.18**	0.0006	0.0009	**-0.21**	0.0012	0.0016	**-0.15**
				(0.15)	(0.17)		(0.0003)	(0.0008)		(0.0003)	(0.0007)		(0.0004)	(0.0010)	
**S2**	410	3001	**0.76**	0.39	0.34	**-0.06**	0.0010	0.0009	**-0.01**	0.0008	0.0008	**0.01**	0.0014	0.0013	**-0.03**
				(0.16)	(0.15)		(0.0003)	(0.0006)		(0.0004)	(0.0005)		(0.0004)	(0.0007)	
**S3**	3763	7269	**-0.32**	0.35	0.41	**-0.08**	0.0009	0.0010	**-0.05**	0.0007	0.0008	**-0.03**	0.0013	0.0015	**-0.08**
				(0.15)	(0.16)		(0.0006)	(0.0006)		(0.0005)	(0.0006)		(0.0007)	(0.0008)	
**S4**	4756	11150	**-0.40**	0.39	0.41	**-0.02**	0.0009	0.0008	**0.04**	0.0007	0.0006	**0.04**	0.0012	0.0012	**0.03**
				(0.15)	(0.15)		(0.0004)	(0.0003)		(0.0004)	(0.0003)		(0.0006)	(0.0004)	
**S5**	938	13791	**0.87**	0.33	0.43	**0.13**	0.0012	0.0008	**-0.21**	0.0010	0.0006	**-0.26**	0.0016	0.0012	**-0.16**
				(0.13)	(0.14)		(0.0007)	(0.0003)		(0.0006)	(0.0003)		(0.0008)	(0.0004)	
**S6**		No data			No data			No data			No data			No data	
**S7**	1796	3951	**-0.37**	0.43	0.44	**-0.01**	0.0008	0.0008	**0.0**	0.0006	0.0006	**0.0**	0.0012	0.0013	**-0.04**
				(0.15)	(0.15)		(0.0003)	(0.0004)		(0.0002)	(0.0003)		(0.0005)	(0.0005)	
**S8**	10899	2326	**0.65**	0.41	0.33	**0.11**	0.0008	0.0011	**-0.16**	0.0006	0.0009	**-0.20**	0.0012	0.0015	**-0.11**
				(0.15)	(0.14)		(0.0003)	(0.0007)		(0.0003)	(0.0007)		(0.0004)	(0.0009)	

B) fODF metric

	AFDtotal			NuFO		
	lCTF	rCTF	AI	lCTF	rCTF	AI
**S1**	0.61<br>(0.29)	0.63<br>(0.40)	**-0.01**	1.45<br>(0.65)	1.28<br>(0.66)	**0.06**
**S2**	0.52<br>(0.39)	0.48<br>(0.27)	**-0.04**	1.25<br>(0.59)	1.61<br>(0.81)	**0.13**
**S3**	0.45<br>(0.27)	0.54<br>(0.25)	**-0.09**	1.82<br>(0.85)	1.49<br>(0.75)	**0.10**
**S4**	0.49<br>(0.29)	0.52<br>(0.28)	**-0.03**	1.71<br>(0.81)	1.80<br>(0.82)	**-0.02**
**S5**	0.40<br>(0.21)	0.51<br>(0.25)	**0.13**	1.47<br>(0.77)	1.70<br>(0.77)	**0.07**
**S6**		No data			No data	
**S7**	0.61<br>(0.36)	0.63<br>(0.34)	**-0.02**	1.41<br>(0.65)	1.33<br>(0.62)	**0.03**
**S8**	0.57<br>(0.29)	0.47<br>(0.31)	**0.10**	1.34<br>(0.64)	1.12<br>(0.64)	**0.09**

C) NODDI metrics

	OD			ICVF			ISOVF		
	lCTF	rCTF	AI	lCTF	rCTF	AI	lCTF	rCTF	AI
**S1**	0.23 (0.13)	0.25 (0.23)	**-0.04**	0.60 (0.13)	0.54 (0.21)	**0.05**	0.12 (0.16)	0.25 (0.30)	**-0.36**
**S2**	0.21 (0.15)	0.33 (0.20)	**0.22**	0.49 (0.20)	0.57 (0.19)	**0.08**	0.17 (0.18)	0.18 (0.27)	**0.05**
**S3**	0.30 (0.18)	0.21 (0.17)	**0.18**	0.53 (0.18)	0.53 (0.20)	**0.00**	0.15 (0.23)	0.19 (0.27)	**-0.14**
**S4**	0.26 (0.16)	0.25 (0.14)	**0.02**	0.57 (0.16)	0.57 (0.13)	**0.00**	0.15 (0.22)	0.12 (0.17)	**0.10**
**S5**	0.27 (0.24)	0.23 (0.14)	**-0.08**	0.44 (0.24)	0.58 (0.13)	**0.14**	0.27 (0.33)	0.11 (0.16)	**-0.43**
**S6**		No data			No data			No data	
**S7**	0.25 (0.14)	0.23 (0.14)	**0.04**	0.59 (0.12)	0.56 (0.15)	**0.03**	0.10 (0.15)	0.13 (0.20)	**-0.13**
**S8**	0.25 (0.13)	0.26 (0.21)	**-0.02**	0.56 (0.12)	0.43 (0.21)	**0.13**	0.12 (0.16)	0.21 (0.28)	**-0.27**

Figure S1. Visualization of all the tracts automatically extract with RecobundleX, visualize with scil_visualize_bundle_mosaic.py (REF). CC and CST are not presented in this mosaic.AC = anterior comissure, AF = arcuate fasciculus, CG = cingulum, FAT = frontal aslant tract, FPT = fronto-pontine tract, FX = fornix, IFOF = inferior fronto-occipital fasciculus, ILF = inferior longitudinal fasciculus, MCP = middle cerebellar peduncle, MdLF = middle longitudinal fascicle, OR_ML = optic radiation and Meyer's loop, PC = posterior commisure, POPT = parieto-occipito pontine tract, SCP = superior cerebellar peduncle, SLF = superior longitudinal fasciculus, UF = Uncinate fasciculus.

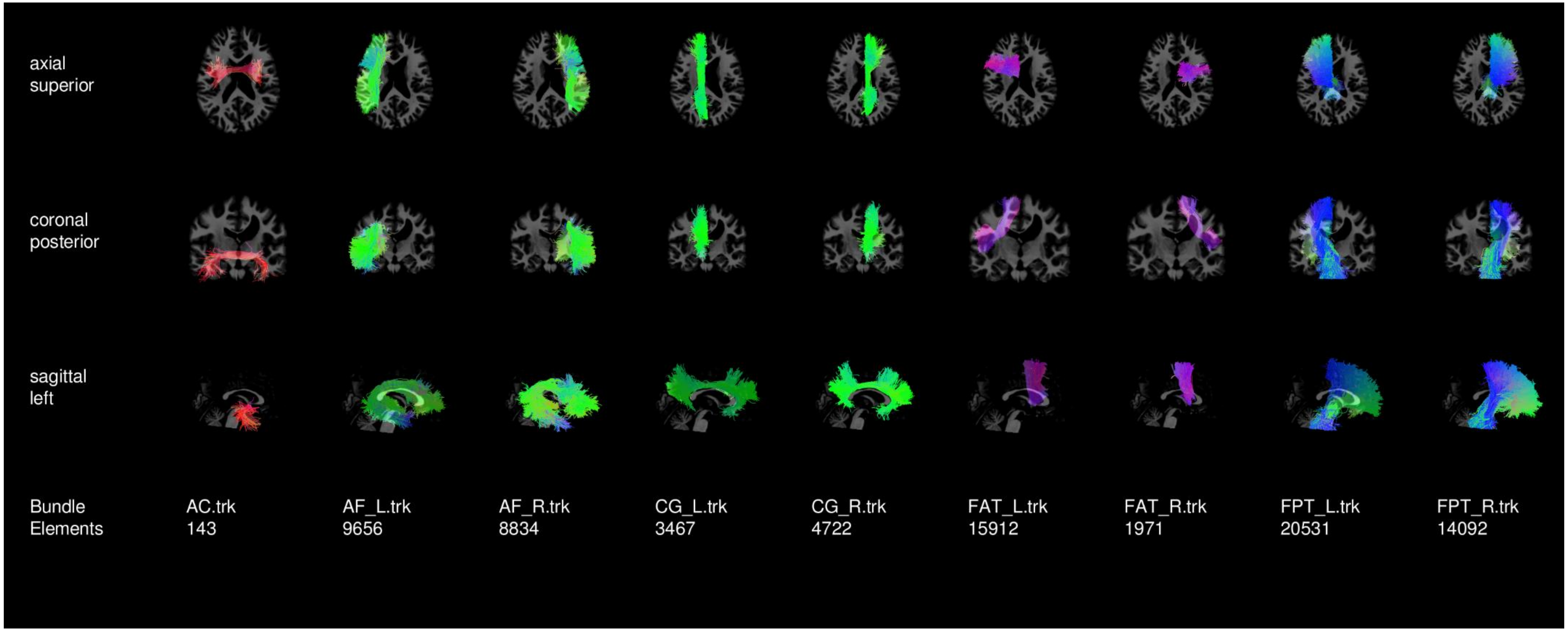

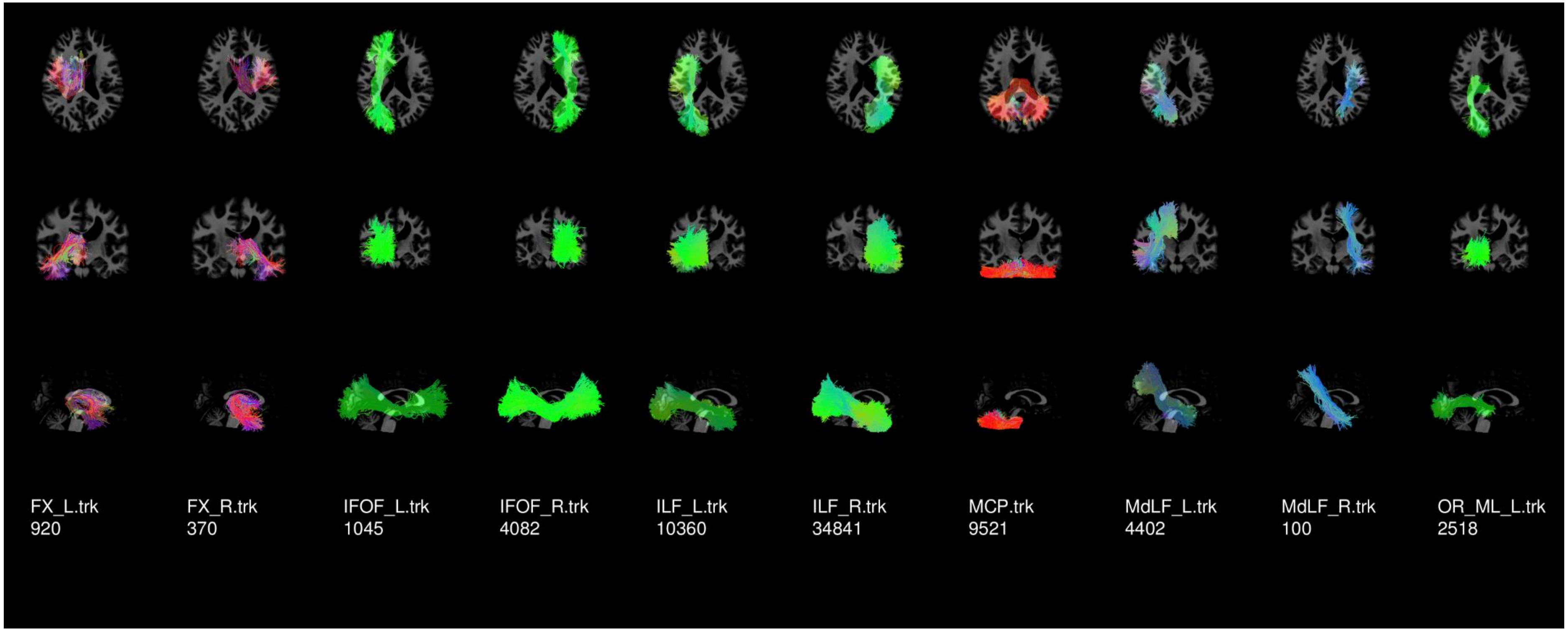
FX_L.trk
920
FX_R.trk
370
IFOF_L.trk
1045
IFOF_R.trk
4082
ILF_L.trk
10360
ILF_R.trk
34841
MCP.trk
9521
MdLF_L.trk
4402
MdLF_R.trk
100
OR_ML_L.trk
2518

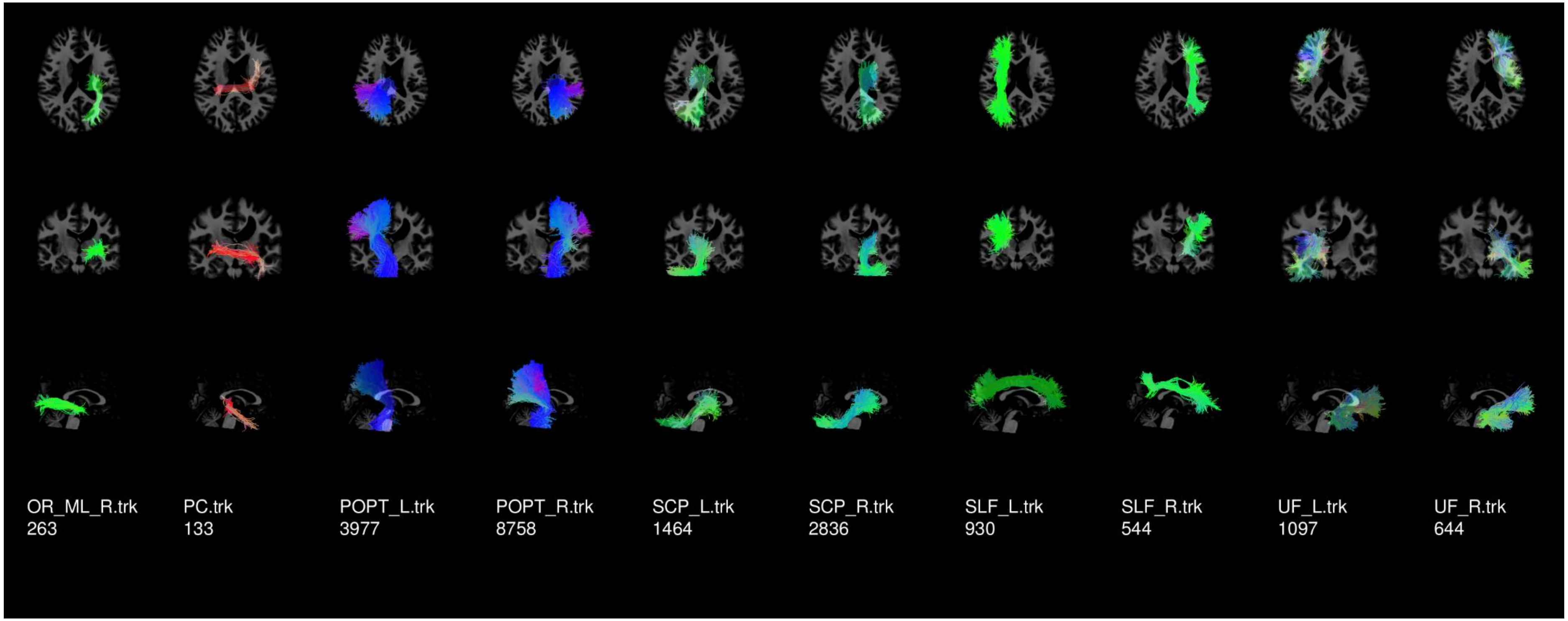
OR_ML_R.trk
263
PC.trk
133
POPT_L.trk
3977
POPT_R.trk
8758
SCP_L.trk
1464
SCP_R.trk
2836
SLF_L.trk
930
SLF_R.trk
544
UF_L.trk
1097
UF_R.trk
644
